# Structural Variations of Bacterial Community Driven by *Sphagnum* Microhabitat Differentiation in a Subalpine Peatland

**DOI:** 10.3389/fmicb.2019.01661

**Published:** 2019-07-24

**Authors:** Wen Tian, Hongmei Wang, Xing Xiang, Ruicheng Wang, Ying Xu

**Affiliations:** ^1^State Key Laboratory of Biogeology and Environmental Geology, China University of Geosciences, Wuhan, China; ^2^Laboratory of Basin Hydrology and Wetland Eco-Restoration, China University of Geosciences, Wuhan, China

**Keywords:** subalpine peatland, *S. palustre*, microhabitat, microbial diversity, water table, total nitrogen

## Abstract

*Sphagnum* microbiomes play an important role in the northern peatland ecosystems. However, information about above and belowground microbiomes related to *Sphagnum* at subtropical area remains largely limited. In this study, microbial communities from *Sphagnum palustre* peat, *S. palustre* green part, and *S. palustre* brown part at the Dajiuhu Peatland, in central China were investigated via 16S rRNA gene amplicon sequencing. Results indicated that Alphaproteobacteria was the dominant class in all samples, and the classes Acidobacteria and Gammaproteobacteria were abundant in *S. palustre* peat and *S. palustre* brown part samples, respectively. In contrast, the class Cyanobacteria dominated in *S. palustre* green part samples. Microhabitat differentiation mainly contributes to structural differences of bacterial microbiome. In the *S. palustre* peat, microbial communities were significantly shaped by water table and total nitrogen content. Our study is a systematical investigation on above and belowground bacterial microbiome in a subalpine *Sphagnum* peatland and the results offer new knowledge about the distribution of bacterial microbiome associated with different microhabitats in subtropical area.

## Introduction

Peatlands only cover approximately 3% of the Earth’s land surface, but store up to ~30% of the terrestrial carbon and thus play a critical role in global carbon cycling (Yu et al., 2010; Mitsch et al., 2013). *Sphagnum*-dominated peatlands are widely distributed in the northern hemisphere (Shaw et al., 2010; Jassey et al., 2011; Weston et al., 2015). *Sphagnum* mosses maintain their dominance through high competition for cation exchange (Soudzilovskaia et al., 2010) and inhibition of growth of other plants attributed to H^+^ release (Weston et al., 2015; Kostka et al., 2016). Due to the dominance and widespread occurrence of *Sphagnum*, the growth of mosses is directly linked to the ecological function of peatland ecosystems.

As the phylogenetically oldest land plant on the Earth, rootless mosses interact with microorganisms (Opelt et al., 2007a) and form highly specific microbiomes (Opelt et al., 2007b; Bragina et al., 2012b, 2015). Recently, *Sphagnum*-associated microbiomes have been demonstrated to greatly affect the growth and health of host mosses (Weston et al., 2015; Kostka et al., 2016). For example, symbiotic methanotrophs and diazotrophs in *Sphagnum* have been confirmed to significantly contribute to CH_4_ consumption in peatlands, CO_2_ and nitrogen supply for *Sphagnum* growth (Kip et al., 2010, 2012; Bragina et al., 2012a, 2013; Berg et al., 2013). Moreover, microbial methane oxidation potential differs among different parts of submerged and non-submerged *Sphagnum* (Raghoebarsing et al., 2005), which may suggest the difference of microbial communities in different parts of *Sphagnum*. Indeed, bacterial 16S rRNA gene copy numbers and community compositions vary between the green part (living part doing the photosynthesis) and the brown part (dying part submerging into the water) of *Sphagnum* (Xiang et al., 2014). These results have stimulated a series of investigation about *Sphagnum* microbiomes (Bragina et al., 2014, 2015) and have expanded our knowledge about the interactions between mosses and microbiomes in peatland ecosystems.

Belowground microbial communities, the drivers of elemental cycling in peatland ecosystems, have also been investigated across different climatic zones, such as high-latitude regions (Belova et al., 2006; Kulichevskaia et al., 2006; Pankratov et al., 2008), tropical rainforest swamps and peatlands (Jackson et al., 2009; Kanokratana et al., 2011; Mishra et al., 2014). A common finding is that microbial communities in peatland ecosystems are mainly composed of several phyla such as Acidobacteria and Proteobacteria (Gilbert and Mitchell, 2006; Andersen et al., 2013). Despite the common dominant taxa in different peatlands, the correlations between microbial communities and environmental factors vary. For instance, water table is demonstrated to influence on the structure of microbial communities (Jaatinen et al., 2007; Kwon et al., 2013; Mishra et al., 2014) or their alpha diversity (Chambers et al., 2016; Urbanová and Barta, 2016; Zhong et al., 2017) in peatlands. pH has been reported to affect soil bacterial communities globally (Bahram et al., 2018) as well as peat microbial communities (Hartman et al., 2008; Lin et al., 2012; Urbanová and Bárta, 2014). Other environmental factors such as temperature and nitrogen content (Pankratov et al., 2008), organic matter content, moisture, and phosphorus (Elliott et al., 2015) also show their impact on microbial communities in peatland ecosystems.

Due to the limited distribution and accessibility of peatlands in subtropical areas, microbiomes remain enigmatic particularly those related to different microhabitats, i.e., different parts of *Sphagnum*. We hypothesize that (1) peatland bacterial microbiome in subtropical region may share the common dominant taxa with other peatland ecosystems but vary among different microhabitats; (2) water table may be one of the most important factors shaping bacterial microbiome in *Sphagnum* peat samples.

To test our hypothesis, the structures of microbial communities from *S. palustre* peat, *S. palustre* brown part, and *S. palustre* green part were investigated via 16S rRNA gene Illumina sequencing and the relationships between bacterial communities and environmental factors were taken into account. The aim of this study was to investigate bacterial communities, especially the above and belowground *Sphagnum*-associated communities in a subtropical peatland. The specific goals were to examine (1) the structure (diversity and composition) of bacterial communities in different microhabitats, and (2) the relationship between peat bacterial communities and environmental factors in the Dajiuhu Peatland.

## Materials and Methods

### Study Area and Sampling

**Figure 1:**
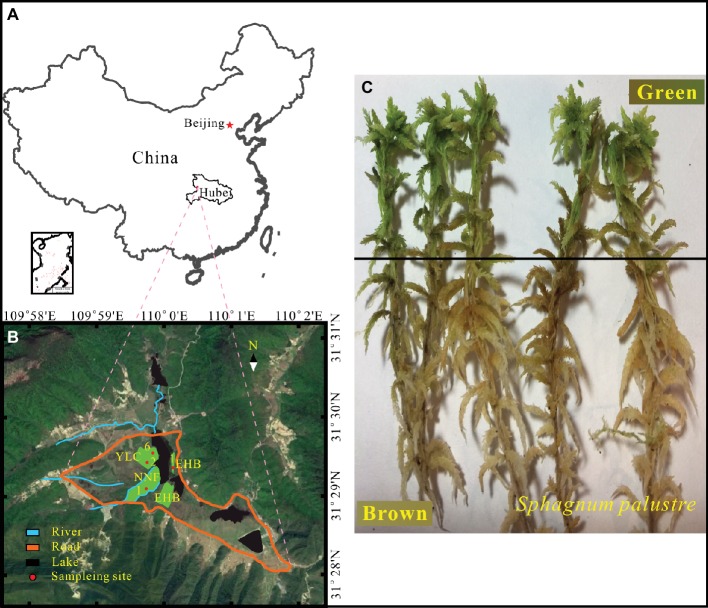
Location of study area **(A)** and sampling sites **(B)** in the Dajiuhu Peatland, Hubei province, central China. **(C)** *S. palustre* brown part and *S. palustre* green part.

The Dajiuhu Peatland (31°24′ ~ 31°33′N, 109°56′ ~ 110°11′E), located in the Shennongjia Forestry District, Hubei province, central China (Figure 1A), is a typical subalpine peatland with a surface area of about 16 km^2^ and 1,730 m a.s.l. The mean annual precipitation and temperature are 1,560 mm and 7.2°C, respectively (Huang et al., 2013). Organic matter is abundant due to the low temperature and high water table (Xiang et al., 2017). Vegetation in the peatland is mainly characterized by *Carex argyi*, *C. capillacea*, *S. palustre*, *Sanguisorba officinalis*, and *Euphorbia esula* with shrubs grown on patches of dry land, which divide the peatland into several small sections. The details of vegetation patterns and hydrological characteristics about the study area have been described previously (Huang et al., 2012; Li et al., 2016).

Samples were collected from four sites in the core area of the Dajiuhu Peatland, which are Erhaoba (EHB), Niangniangfen (NNF), and two sites at Yangluchang (YLC) with different water table (Figure 1B) in July 2016. Water table is expressed with negative number when it is below the peat surface and *vice versa*. At each site, *S. palustre* peat (0–5 cm) and *S. palustre* were collected in triplicates. Underlying *S. palustre* peat samples were stored in 50-ml sterile centrifuge tubes and *S. palustre* samples were placed in sterile plastic bags. All samples were transported to the geomicrobiology laboratory at China University of Geosciences (Wuhan) on dry ice within 12 h. *S. palustre* brown part and *S. palustre* green part were cut off aseptically according the color identification in the laboratory. In total, 36 samples [3 replicates × 3 microhabitats (*S. palustre* peat, *S. palustre* brown part, and *S. palustre* green part) × 4 sites] were examined. Visible plant roots, litter, and debris were removed from peat samples. Half of each peat sample was stored at 4°C for chemical analysis and the other half was stored at −20°C for DNA extraction.

### Physicochemical Analysis

*S. palustre* peat samples (approx. 20 g) were dried at 50°C for 24 h to measure the water content. One half of each dried peat sample was ground to 200-mesh after fumigation with concentrated HCl (Horwáth and Kessel, 2001), and analyzed for total organic carbon (TOC) and total nitrogen (TN) with a Vario EL III Elemental Analyzer (Elementar, Germany). The other half was converted to ash at 550°C for 3 h to measure the organic matter content. Peat samples weighing 5 g were sonicated in deionized water (1:4 w/v ratio) for 15 min, followed by 20 min of shaking at 200 rpm. The suspension was centrifuged at 5,000 g for 5 min (Elliott et al., 2015) and the pH of the supernatant was measured with a multi-parameter water quality detector (HACH, Loveland, CO). The supernatant was filtered through 0.22-μm membrane and the filtrate was analyzed for major anions and cations using an ICS-600 ion chromatograph (Yun et al., 2016).

### DNA Extraction, PCR, and Illumina Sequencing

*S. palustre* samples were cut into two parts (Figure 1C) according to color identification and were designated as SB for *S. palustre* brown part and SG for *S. palustre* green part, respectively. The samples were rinsed three times with sterile distilled water for 5 min each time to remove adhesive debris before DNA extraction. Approximately 0.5 g of frozen-dried samples were subjected to DNA extraction with the PowerSoil DNA Isolation Kit (QIAGEN, Düsseldorf, NW) following the manufacturer’s instruction (Xiang et al., 2014). Total DNA was quantified using a NanoDrop 2000 spectrophotometer (Thermo Fisher Scientific, Waltham, MA) and 1% agarose gel was used to check the DNA quality. The V3 and V4 regions of microbial 16S rRNA were amplified using the primers 347F (5′-CCTACGGRRBGCASCAGKVRVGAAT-3′) and 802R (5′-GGACTACNVGGGTWTCTAATCC-3′) (Cai et al., 2019). After amplification, PCR products were purified and quantified by Qubit 2.0 Fluorometer (Invitrogen, Carlsbad, CA). DNA libraries were validated by Agilent 2100 Bioanalyzer (Agilent Technologies, Palo Alto, CA), and then applied to Illumina MiSeq sequencing with 2 × 300 bp paired-end at GENEWIZ, Inc. (Suzhou, China).

### Data Processing

The raw sequences obtained from MiSeq reads were demultiplexed through the barcode sequences and processed with Quantitative Insight Into Microbial Ecology (QIIME 1.9.1 version) pipeline (Caporaso et al., 2010). The sequences of an individual sample were identified based on their unique barcode, which subsequently went through the removal of primers and barcodes, and paired-end assembly. The high-quality sequences were determined based on (1) absence of any ambiguous base “N”, (2) length > 200 bp, and (3) base quality score > 20 in a sliding window. The chloroplast was removed and the chimeric sequences were identified using the UCHIME algorithm (Edgar et al., 2011). The clean sequences were classified into operational taxonomic units (OTUs) by UCLUST (Edgar, 2010) based on 97% sequence similarity and singletons (OTUs with only one sequence in all samples) were filtered out. Taxonomic category assignment was conducted with representative sequences at a threshold of 0.8 in the Ribosomal Database Program classifier (Cole et al., 2009), followed by annotation with SILVA 119 database. All the samples were resampled to the same number of reads (29,549) using mothur (Schloss et al., 2009) prior to statistical analysis.

The original 16S rRNA sequence data have been deposited in the NCBI Sequence Read Archive^1^ under the accession number PRJNA512496.

### Statistical Analysis

One-way analysis of variance (ANOVA) was used to test the microhabitat (*S. palustre* peat, *S. palustre* brown part, and *S. palustre* green part) impact on alpha diversity and the relative abundance of different taxa in SPSS 18.0. Principal coordinate analysis (PCoA) based on Bray-Curtis distance metrics was conducted to visualize the community dissimilarity among the samples and recognize key factors driving variations of community structure. Permutational multivariate analysis of variance (PERMANOVA) with the Bray-Curtis and Jaccard distances matrixes was used to compare the dissimilarity degree of community among groups. PCoA coupled with PERMANOVA quantified the differences of *S. palustre* peat communities based on Bray-Curtis distances. Beta diversity analysis was performed using “vegan” package in R (v. 3.4.1). The indicator species in different microhabitats were identified via linear discriminant analysis effect size (LEfSe) (Segata et al., 2011). Regression model between water table and alpha diversity in *S. palustre* peat was performed in R (v. 3.4.1) “basicTrendline” package. The correlations between environmental factors and bacterial communities were revealed by redundancy analysis (RDA) using Canoco 5 (Braak and Šmilauer, 2015).

## Results

### Geochemistry of *S. palustre* Peat

**Figure 2:**
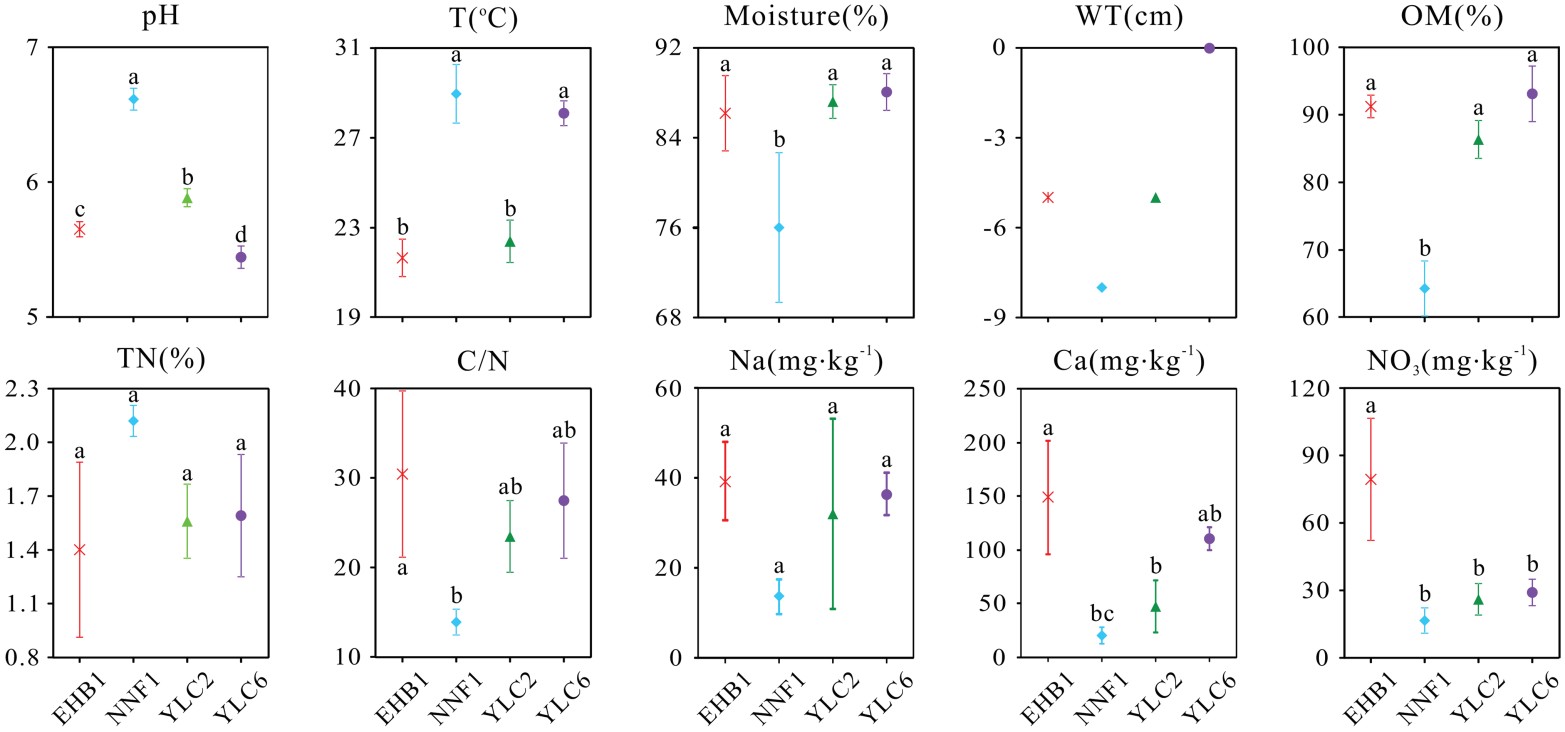
Physicochemical properties of *S. palustre* peat samples in the Dajiuhu Peatland. WT, water table; OM, organic matter; TN, total nitrogen; C/N, the ratio of total organic carbon to total nitrogen. EHB1, the first site of Erhaoba; NNF1, the first site of Niangniangfen; YLC2, the second site of Yangluchang; YLC6, the sixth site of Yangluchang. Negative values of WT represent belowground levels. Bars indicate the standard deviation (*n* = 3) except WT. Lowercase letters represent significant differences at 95% confident interval as indicated by ANOVA.

The geochemical properties of *S. palustre* peat samples are listed in Supplementary Table S1. All peat samples were acidic, with pH ranging from 5.35 to 6.70, and significant differences were observed (*α* = 0.05) among the four sampling sites (Figure 2). Overall, the moisture and organic matter content (OM) were high (69.16–89.95% and 60.87–97.72%, respectively), and total nitrogen (TN) was low (1.03–2.17%) in all samples. Concentrations of ${\mathrm{NO}}_{3}^{-}$, Na^+^, and Ca^2+^ were significantly higher in samples at the first site of Erhaoba (EHB1) (mean 79.29 mg kg^−1^ ${\mathrm{NO}}_{3}^{-}$, 39.26 mg kg^−1^ Na^+^, and 148.84 mg kg^−1^ Ca^2+^) than those of samples at the first site of Niangniangfen (NNF1) (mean 16.56 mg kg^−1^ ${\mathrm{NO}}_{3}^{-}$, 13.59 mg kg^−1^ Na^+^, and 15.60 mg kg^−1^ Ca^2+^). Temperature (23.1–30.3°C) and water table (−8 to 0 cm) varied among *S. palustre* peat samples.

### Alpha and Beta Diversities of Bacterial Microbiome

**Figure 3:**
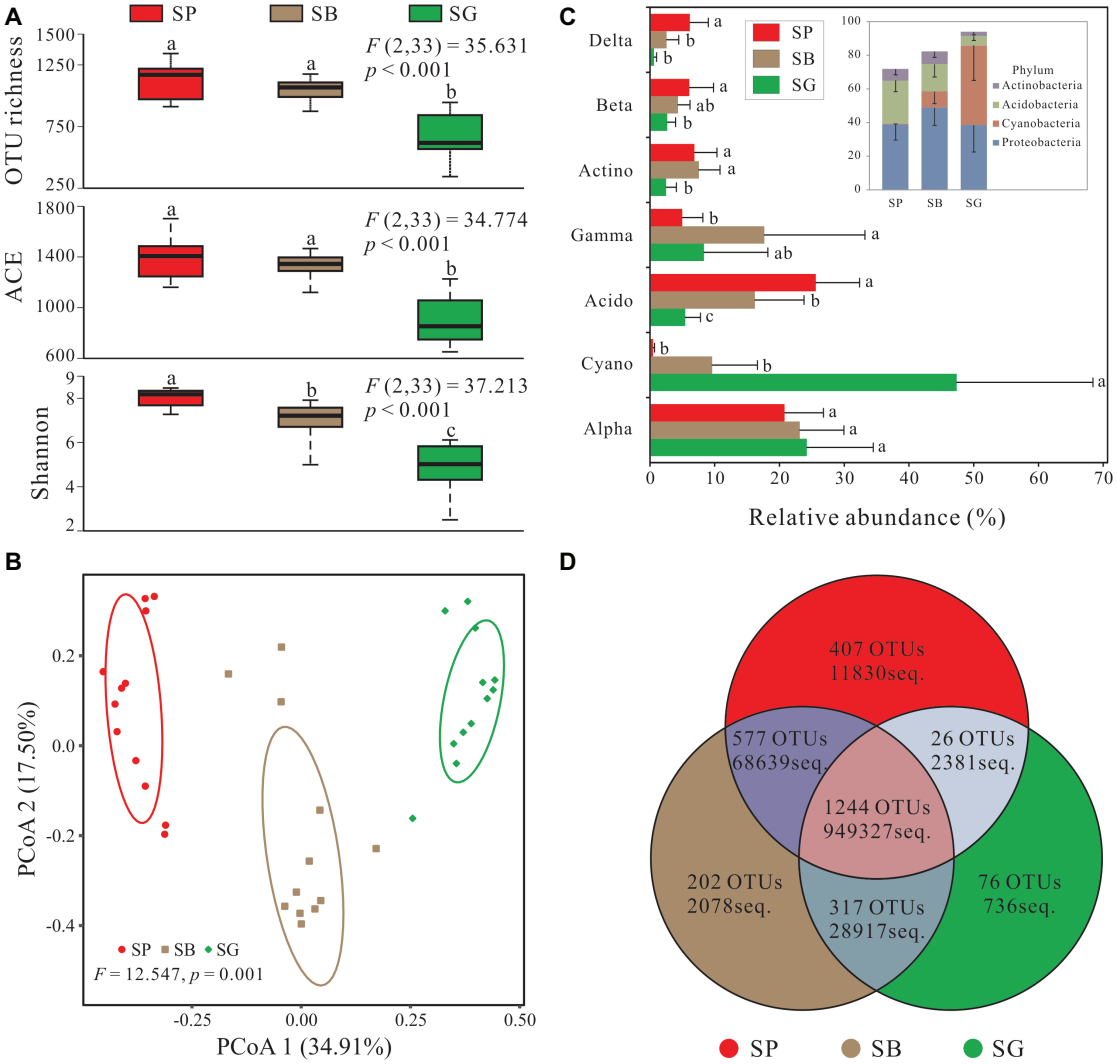
*Sphagnum*-associated bacterial communities. **(A)** Alpha diversity of 16S rRNA genes in different microhabitats. Box plots show the maximum and minimum, median, first (25%) and third (75%) quartiles observed values in each dataset (*n* = 12). Data were analyzed by the one-way ANOVA with Tukey’s HSD *post hoc* comparisons. The test statistical values of F (DFn, DFd) are shown at the right top of each graph. Significant differences (*p* < 0.05) across groups are indicted with lowercase letters. **(B)** The principal coordinate analysis (PCoA) plots based on the weighted Bray-Curtis metrics among samples. Ellipses indicate 95% confidence level. **(C)** Dominant phyla (relative abundance >5%) and corresponding classes’ distribution in different microhabitats. **(D)** Numbers of mutual OTUs and unique OTUs in each microhabitat. Unclassified and archaea OTUs are removed. Acido, Acidobacteria; Actino, Actinobacteria; Alpha, Alphaproteobacteria; Beta, Betaproteobacteria; Cyano, Cyanobacteria; Delta, Deltaproteobacteria; Gamma, Gammaproteobacteria. SP, *S. palustre* peat; SB, *S. palustre* brown part; SG, *S. palustre* green part.

In total, 2,849 OTUs were recovered after quality control and resampled (archaea and unclassified excluded) (Supplementary Table S2). Alpha diversity was the highest in *S. palustre* peat samples, followed by *S. palustre* brown part. The *S. palustre* green part showed the lowest alpha diversity as indicated by OTU richness, ACE index, and Shannon index (Figure 3A). Shannon diversity of bacterial communities was statistically different between *S. palustre* peat and *S. palustre* (*α* = 0.05), and between *S. palustre* brown part and *S. palustre* green part (*α* = 0.05).

PCoA revealed a clear clustering pattern of bacterial communities at the OTU level according to different microhabitats of *S. palustre*. PCoA1 and PCoA2 in Bray-Curtis matrix explained 34.91% and 17.50% of the variance, respectively (Figure 3B). Bacterial communities between any two microhabitats were significantly (*α* = 0.05) different as indicated by PERMANOVA both at the OTU and phylum levels. However, fewer differences were observed among sampling sites (Table 1).

**Table 1:** PERMANOVA indicating dissimilarities of bacterial community among different microhabitats and sampling sites.

Distance	Bray-Curtis				Jaccard			
Phylogenetic level	OTU		Phylum		OTU		Phylum	
PERMANOVA output	*F*	*p*	*F*	*p*	*F*	*p*	*F*	*p*
Microhabitat								
SP vs. SB	7.56	0.003^**^	7.738	0.003^**^	4.980	0.003^**^	5.964	0.003^**^
SP vs. SG	21.65	0.003^**^	32.163	0.003^**^	11.813	0.003^**^	21.248	0.003^**^
SB vs. SG	9.988	0.003^**^	21.511	0.003^**^	6.572	0.003^**^	14.184	0.003^**^
Sampling site								
EHB1 vs. NNF1	1.006	1.000	0.018	1.000	1.151	1.000	0.171	1.000
EHB1 vs. YLC2	0.954	1.000	2.045	0.798	1.067	1.000	1.912	0.696
EHB1 vs. YLC6	3.077	0.126	2.778	0.390	2.489	0.114	2.530	0.312
NNF1 vs. YLC2	1.269	1.000	3.020	0.408	1.344	1.000	2.614	0.378
NNF1 vs. YLC6	3.109	0.108	2.994	0.372	2.671	0.048^*^	2.961	0.222
YLC2 vs. YLC6	3.081	0.036^*^	4.967	0.042^*^	2.497	0.066	4.474	0.018^*^

*PERMANOVA, permutational multivariate analysis of variance; SP, S. palustre peat; SB, S. palustre brown part; SG, S. palustre green part; EHB1, the first site of Erhaoba; NNF1, the first site of Niangniangfen; YLC2, the second site of Yangluchang; YLC6, the sixth site of Yangluchang. F, test statistic; *p*’s are corrected by false discovery rate (FDR)*.

* *p ≤ 0.05*,

** *p ≤ 0.01*.

### Taxonomic Composition of Bacterial Microbiome

In total, 30 bacterial phyla and 41 classes were identified across all samples, and dominant taxa varied among different microhabitats (Figure 3C). In *S. palustre* peat samples, the majority of reads were assigned to Acidobacteria and Alphaproteobacteria. While in *S. palustre* brown part samples, Alphaproteobacteria and Gammaproteobacteria were the dominant taxa. In *S. palustre* green part, Cyanobacteria and Alphaproteobacteria dominated the microbial communities. The relative abundance of Alphaproteobacteria and Cyanobacteria decreased from SG (24.21 ± 10.28 and 47.36 ± 21.05%) to SB (23.14 ± 6.82 and 9.61 ± 6.98%) and further to SP (20.79 ± 6.00 and 0.49 ± 0.22%) (Supplementary Table S3). In contrast, Acidobacteria, Betaproteobacteria, and Deltaproteobacteria showed the opposite trend, which significantly increased from SG (5.45 ± 2.35, 2.68 ± 1.27% and 0.62 ± 0.40%) to SB (16.20 ± 7.59, 4.32 ± 1.85 and 2.57 ± 1.89%) and further to SP (25.62 ± 6.76, 6.08 ± 3.74 and 6.17 ± 2.84%). Acidobacteria and Deltaproteobacteria (ANOVA, *p* < 0.05) were significantly enriched in *S. palustre* peat samples. While in *S. palustre* green part samples, Cyanobacteria (ANOVA, *p* < 0.05) was significantly different compared to that in *S. palustre* peat and *S. palustre* brown part.

### Indicator Groups for Different Microhabitats

The overview of OTU distribution among different microhabitats showed that 1,244 OTUs were shared by three microhabitats (Figure 3D). *S. palustre* peat and *S. palustre* brown part shared the most OTUs (1,821), followed by that in *S. palustre* brown and green parts (1,561). The least OTUs (1,270) were shared between *S. palustre* peat and *S. palustre* green part. About 407 of the total OTUs were exclusively observed in *S. palustre* peat as compared to *S. palustre* brown part (202 OTUs) and *S. palustre* green part (76 OTUs) (Figure 3D).

**Figure 4:**
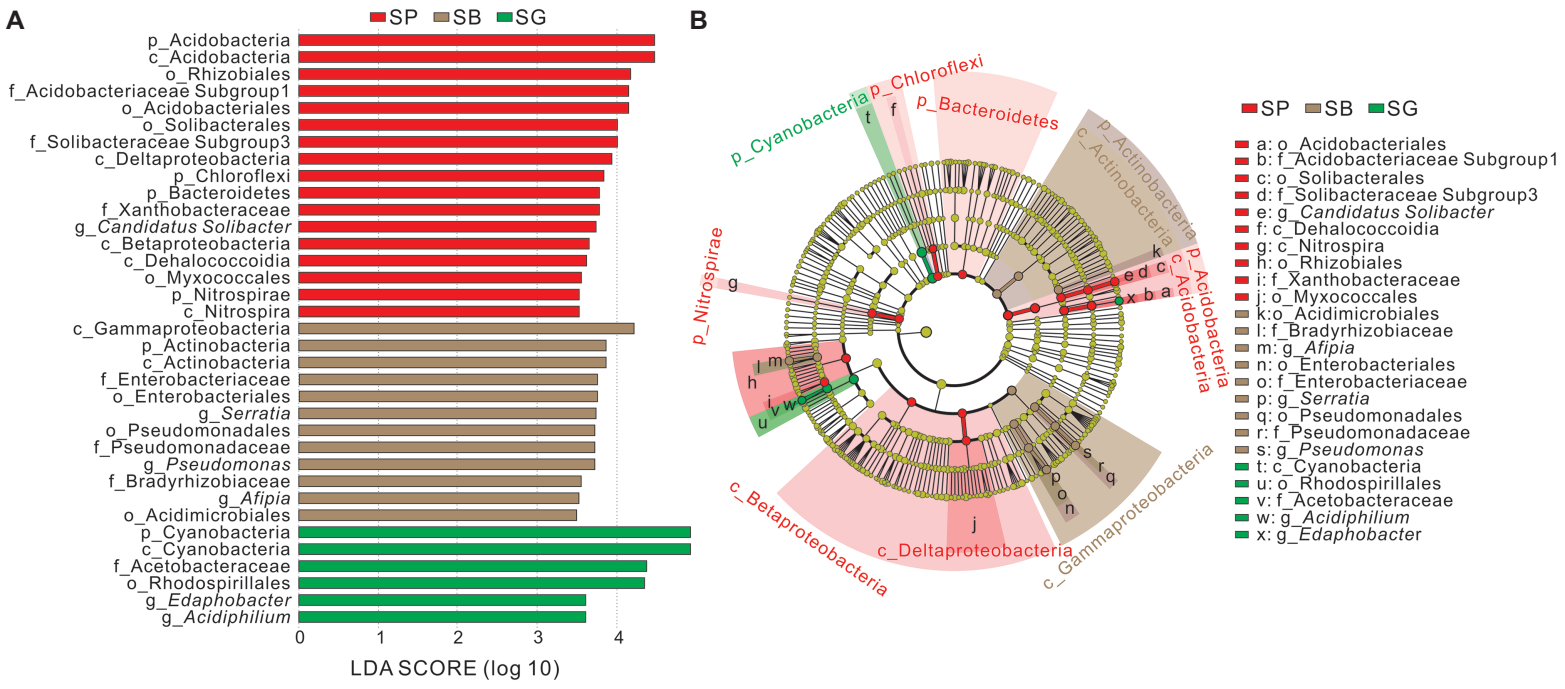
Indicator groups analysis of bacterial communities in different microhabitats of *S. palustre* with LDA SCORE > 3.5 **(A)** and taxonomic cladogram **(B)** through linear discriminant analysis effect size (LEfSe). Nodes from inside to outside represent the phylogenetic levels from phylum to genus, respectively. Yellow nodes represent taxa that do not significantly discriminate among microhabitats. Significant discriminant taxa of *S. palustre* peat, *S. palustre* brown part, and *S. palustre* green part are highlighted in red, brown, and green, separately. The dimension of nodes is positively correlated with the relative abundance of taxon. Abbreviations are described in Figure 3.

The LEfSe analysis was conducted to identify indicator groups in different microhabitats (Figure 4). In *S. palustre* peat samples, 17 indicator species were identified which belonged to the phyla Acidobacteria, Bacteroidetes, Chloroflexi, and Nitrospirae, classes Acidobacteria, Dehalococcoidia, Nitrospira, Betaproteobacteria and Deltaproteobacteria. The indicator genus was *Candidatus Solibacter* in peat samples. In *S. palustre* brown part samples, 12 indicator species were enriched which were affiliated with the phylum Actinobacteria, classes Actinobacteria and Gammaproteobacteria. *Afipia*, *Serratia*, and *Pseudomonas* were indicator genera in the *S. palustre* brown part. In *S. palustre* green part samples, six indicator groups were enriched which fell into the phylum Cyanobacteria, class Cyanobacteria, order Rhodospirillales, family Acetobacteraceae, and genera *Edaphobacter* and *Acidiphilium*.

### Correlations Between Physicochemical Characteristics and *S. palustre* Peat Bacterial Communities

**Figure 5:**
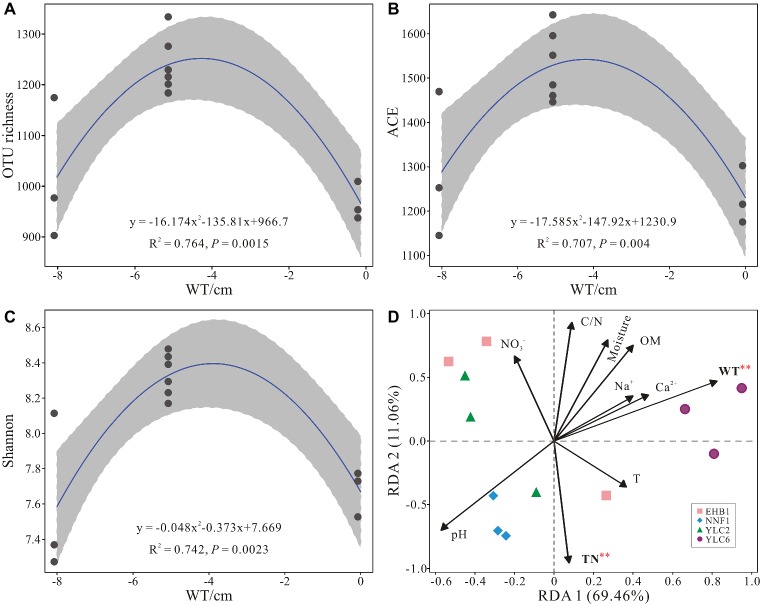
Relationship between water table and alpha diversity of bacterial communities from *S. palustre* peat samples **(A–C)** and redundancy analysis showing the relationships between environmental factors and bacterial communities from *S. palustre* peat samples **(D)**. Significant levels (*p* < 0.01) are marked with red asterisk based on permutation test (*n* = 1,000). Abbreviations of environmental factors and sampling site are the same as those in Figure 2.

Alpha diversity (OTU richness, ACE and Shannon indices) showed a significant correlation with water table as indicated by regression models (*p* < 0.05) (Figures 5A–C). Water table and total nitrogen were found to significantly affect microbial communities in *S. palustre* peat samples as indicated by RDA analysis (Figure 5D), which explained 51.3% and 22.1% of the variation, respectively. Bacterial communities from the NNF1 were positively correlated with total nitrogen and negatively correlated with water table, whereas those from the sixth site of Yangluchang (YLC6) were positively correlated with water table (Figure 5D). Specifically, Bacteroidetes (*r* = 0.922, *p* < 0.01) and Chloroflexi (*r* = 0.683, *p* < 0.05) were positively correlated with water table, whereas Acidobacteria (*r* = −0.751, *p* < 0.01) and Actinobacteria (*r* = −0.751, *p* < 0.01) were negatively correlated with water table (Supplementary Table S4). Bacteroidetes (*r* = −0.613, *p* < 0.05) were negatively correlated with total nitrogen (Supplementary Table S4). Briefly, the structure of bacterial communities in *S. palustre* peat altered from low water table NNF1 (−8 cm) to high water table YLC6 (0 cm) (Supplementary Figure S1 and Supplementary Table S5).

## Discussion

### Variation of Bacterial Microbiome Among Microhabitats

Generally, bacterial communities are mainly composed of several dominant phyla in a wide range of soil types as well as in peatlands, e.g., Proteobacteria, Acidobacteria, Bacteroidetes, Actinobacteria, and Firmicutes (Griffiths et al., 2016; Karimi et al., 2018; Shi et al., 2018). Our results with Proteobacteria and Acidobacteria as the dominant peat microbial taxa were consistent with those in other peatlands (Kraigher et al., 2006; Ausec et al., 2009; Danilova et al., 2016).

Alpha diversity changed across microhabitats in our study. For example, a remarkable loss of alpha diversity (e.g., Shannon index from 7.02 in SB to 4.78 in SG) and decrease in total and specific OTUs (from 2,340 in SB to 1,633 in SG, and 202 in SB to 76 in SG, respectively) (Figures 3A,D) were observed from *S. palustre* brown part to *S. palustre* green part which clearly showed a microhabitat differentiation. This differentiation was also confirmed by the clustering of community structure based on PCoA (Figure 3B).

These changes may closely relate to the specific cell types in green part and brown part of *Sphagnum*. *Sphagnum* green part consists of live photosynthetic cells (chlorocytes) and *Sphagnum* brown part composes of dead and water-filled hyaline cells (hyalocytes). Hyalocytes allow free movement of solutes or microorganisms in and out (Raghoebarsing et al., 2005; Shcherbakov et al., 2013), and further resulted in higher biodiversity of microbial communities in *Sphagnum* brown part. In contrast, microbial colonization in *Sphagnum* green part is strictly selected by chlorocytes (Opelt et al., 2007b; Bragina et al., 2012b), thus decreased microbial community diversity.

Moreover, the difference in the trophic status may also lead to the changes of bacterial microbiome in different microhabitats. The ratio of Proteobacteria to Acidobacteria has been used as an indicator of nutrient status in soil ecosystems (Smit et al., 2001) as well as in different peatlands (Hartman et al., 2008; Urbanová and Bárta, 2014). As a cosmopolitan taxon in acidic environments, Acidobacteria is able to grow under oligotrophic conditions (Philippot et al., 2010; Dedysh, 2011; Andersen et al., 2013). Proteobacteria are usually associated with higher carbon availability according to the copiotroph-oligotroph theory (Fierer et al., 2007; Leff et al., 2015). Thus, a higher ratio of Proteobacteria to Acidobacteria would indicate nutrient-rich conditions, or *vice versa*. Generally, species richness and microbial diversity increase with the improvement of nutritional status in peat sediments (Urbanová and Bárta, 2014). In our study, the ratio of Proteobacteria to Acidobacteria was 1.52 ± 0.38, 3.02 ± 1.03, and 7.08 ± 1.78 in *S. palustre* peat, *S. palustre* brown part, and *S. palustre* green part, respectively, indicating a relative nutrient-poor condition in *Sphagnum* peat compared with those in *Sphagnum* mosses. However, bacterial diversity decreased from *S. palustre* peat to *S. palustre* green part. This discrepancy was also observed previously (Xiang et al., 2014), which may result from the strict selection to microbiomes by mosses.

Besides variations in microbial diversity across microhabitats, indicator taxa also varied (Figure 4), which might indicate the microbial preference to specific microhabitats and the variation in microbial potential functions. *Candidatus Solibacter* affiliated with Acidobacteria are the indicator taxa in *S. palustre* peat, which were reported to be capable of utilizing various carbon sources, and reducing nitrate and nitrite under oligotrophic conditions (Ward et al., 2009) and further adapt to the acidic peatlands (Kanokratana et al., 2011). In this study, all the peat samples were acidic (pH: 5.35–6.70) with a low content of total nitrogen (1.03–2.17%), which may favor the growth of *Candidatus Solibacter* and thus result in their dominance. *Serratia* and *Pseudomonas* belonging to Gammaproteobacteria are the indicator groups in *S. palustre* brown part. *Serratia* were reported to be typical dwellers of *Sphagnum* plants in peat bogs (Opelt and Berg, 2004; Belova et al., 2006) and the major antagonists in *S. fallax* (Opelt et al., 2007c). Moreover, the isolated endophytic bacteria with high antagonistic potential were also confirmed to be *Serratia* and *Pseudomonas* in *Sphagnum* mosses (Shcherbakov et al., 2013). Therefore the dominance of *Serratia* and *Pseudomonas* in *S. palustre* brown part may associate the antagonistic potential of *Sphagnum*. *Acidiphilium* and *Edaphobacter* are the indicator species in *S. palustre* green part. *Acidiphilium* affiliated with Alphaproteobacteria are obligate acidophilic chemoorganotrophic bacteria (Okamura et al., 2015) and produce zinc-bacteriochlorophyll (Zn-BChl) a only under aerobic conditions (Hiraishi and Shimada, 2001). Compared with Mg-BChl a, Zn-BChl a is more stable and has advantages in photosynthesis under acidic environments. *Edaphobacter* are obligate aerobic and heterotrophic organisms, which may prefer neutral to slightly acidic environments and be isolated from a co-culture with a methanotrophic bacterium (Koch et al., 2008). Hence, the enriched *Acidiphilium* and *Edaphobacter* in *S. palustre* green part may benefit the growth of *Sphagnum*.

### Water Table and Total Nitrogen Shape Bacterial Communities in *S. palustre* Peat

Multiple environmental factors have been demonstrated to affect peat microbial communities, e.g., water table (Kwon et al., 2013; Mishra et al., 2014; Chambers et al., 2016; Urbanová and Barta, 2016; Zhong et al., 2017), pH (Hartman et al., 2008; Lin et al., 2012), temperature (Pankratov et al., 2011), organic matter (Elliott et al., 2015), and C/P ratio (Lin et al., 2014).

Our results showed that the decrease of water table altered alpha diversity of bacterial communities (OTU richness, ACE and Shannon indices) in *S. palustre* peat. Alpha diversity significantly (regression models, *p* < 0.05) correlated with water table (Figures 5A–C). These changes of community diversity were probably related to the differences in peat physicochemical properties (Lauber et al., 2009; Zhong et al., 2017). In our study, the decrease of TOC and increase of TN content with water table decrease supported the perspective (Supplementary Table S1) and matched with observations reported from other peatlands (Urbanová and Bárta, 2014; Urbanová and Barta, 2016). Interestingly, the alpha diversity presented a unimodal correlation with water table change and higher value was observed at the oxic-anoxic interfaces (−5 cm). Microbial activities and diversity could be enhanced at the oxic-anoxic interface, in which the alteration of electron donors and acceptors often happens with the fluctuation of water table (Brune et al., 2000; Daffonchio et al., 2006). Besides the changes in alpha diversity, water table also significantly altered the composition and structure of bacterial communities (Supplementary Figure S1). The decrease in water table could influence community structure via several ways: altering the oxic-anoxic interface, increasing peat decomposability, and the bulk density of surface peat (Jaatinen et al., 2007; Urbanová and Barta, 2016). In our study, with the decrease of water table from YLC6 (0 cm) to EHB1 (−5 cm) or YLC2 (−5 cm) to NNF1 (−8 cm), more oxygen could penetrate into *Sphagnum* peat and increase the thickness of aerobic layer, which leads to the increase of the relative abundance of Actinobacteria, Alphaproteobacteria, and Gammaproteobacteria, and decrease of the relative abundance of Deltaproteobacteria and Chloroflexi (Supplementary Table S5). Previous studies also show that with the decrease of water table, the relative abundance of Alphaproteobacteria and Gammaproteobacteria increases in a tropical peatland (Mishra et al., 2014), whereas Deltaproteobacteria decrease in bogs and fens (Urbanová and Barta, 2016). These results are consistent with ours in the Dajiuhu Peatland. Furthermore, the variation of water table affects microbial functional groups. For example, heterotrophic Actinobacteria can degrade recalcitrant polymeric substances such as lignin, chitin, pectin, aromatics, and humic acids (Killham and Prosser, 2015) under aerobic conditions and thus thrive in oxic layers in acidic peatlands (Jaatinen et al., 2007). Lower water table also significantly increased the relative abundance of aerobic Methylocystaceae (methanotrophs) (Supplementary Table S5), which may further impact CH_4_ emission in peatland ecosystems (Kwon et al., 2013; Zhong et al., 2017). In contrast, anaerobes such as Chloroflexi favor a higher water table.

Total nitrogen also significantly affected peat bacterial communities. Nitrogen is usually limited in boreal peatlands, especially *Sphagnum*-dominated peatlands (Wu and Blodau, 2013; Vile et al., 2014). *Sphagnum* mosses can obtain nitrogen via their symbiotic microbes, e.g., *Bradyrhizobium*, *Beijerinckia*, *Burkholderia*, *Pseudomonas*, *Sphingobacterium*, and methanotrophs (*Methylobacterium* and *Methylocella*) as well as Cyanobacteria (Bragina et al., 2012a, 2013). After death, *Sphagnum* mosses can serve as important N-sources in peatland ecosystems, which can be converted to different N forms, e.g., NH_4_^+^ (van den Elzen et al., 2017) under aerobic conditions, thus increasing the bioavailability of nitrogen (Rütting et al., 2011). In contrast, anaerobic conditions resulting from higher water table would lead to the rapid immobilization of nitrogen, thus decreasing microbial activity (Zhang et al., 2018).

## Conclusion

In the present study, we confirmed that bacterial microbiome in the Dajiuhu Peatland, a subtropical peatland, shared the dominant bacterial phyla such as Proteobacteria and Acidobacteria with other peatlands. The microhabitats were found to play a fundamental role in the structure of bacterial microbiome from *S. palustre* peat to *S. palustre* brown part and further to *S. palustre* green part. Community richness and alpha diversity were the highest in *S. palustre* peat and the lowest in *S. palustre* green part. Distinctive indicator groups identified in each microhabitat also supported our hypothesis about microhabitat differentiation driving the variations of bacterial microbiome. Cell types in different parts of *Sphagnum* may account for the difference in their bacterial microbiome. Water table and total nitrogen shaped the bacterial communities in *S. palustre* peat. These results enhance our understanding about microbial variation among microhabitats in subalpine peatland ecosystems. However, microbial function variations across different microhabitats needed to be investigated in near future.

## Data Availability

The datasets generated for this study can be found in National Center for Biotechnology Information (NCBI), PRJNA512496.

## Author Contributions

WT conducted the experiments, analyzed data, and drafted the manuscript. HW designed the study, provided the funding, and revised the manuscript. XX helped with data analyses. RW and YX helped with sample collection.

### Conflict of Interest Statement

The authors declare that the research was conducted in the absence of any commercial or financial relationships that could be construed as a potential conflict of interest.

## Footnotes

^1^https://www.ncbi.nlm.nih.gov/sra

## References

[ref1] Andersen R. Chapman S. J. Artz R. R. E. (2013). Microbial communities in natural and disturbed peatlands: a review. Soil Biol. Biochem. 57, 979–994. 10.1016/j.soilbio.2012.10.003

[ref2] Ausec L. Kraigher B. Mandic-Mulec I. (2009). Differences in the activity and bacterial community structure of drained grasslandand forest peat soils. Soil Biol. Biochem. 41, 1874–1881. 10.1016/j.soilbio.2009.06.010

[ref3] Bahram M. Hildebrand F. Forslund S. Anderson J. Soudzilovskaia N. A. Bodegom P.. (2018). Structure and function of the global topsoil microbiome. Nature 560, 233–237. 10.1038/s41586-018-0386-6, PMID: 30069051

[ref4] Belova S. E. Pankratov T. A. Dedysh S. N. (2006). Bacteria of the genus *Burkholderia* as a typical component of the microbial community of *Sphagnum* peat bogs. Microbiology 75, 110–117. 10.1134/s0026261706010164 16579452

[ref5] Berg A. Danielsson A. Svensson B. H. (2013). Transfer of fixed-N from N_2_-fixing Cyanobacteria associated with the moss *Sphagnum riparium* results in enhanced growth of the moss. Plant Soil 362, 271–278. 10.1007/s11104-012-1278-4

[ref6] Braak C. J. F. T. Šmilauer P. (2015). Topics in constrained and unconstrained ordination. Plant Ecol. 216, 1–14. 10.1007/s11258-014-0356-5

[ref7] Bragina A. Berg C. Berg G. (2015). The core microbiome bonds the Alpine bog vegetation to a transkingdom metacommunity. Mol. Ecol. 24, 4795–4807. 10.1111/mec.13342, PMID: 26335913

[ref8] Bragina A. Berg C. Cardinale M. Shcherbakov A. Chebotar V. Berg G. (2012b). *Sphagnum* mosses harbour highly specific bacterial diversity during their whole lifecycle. ISME J. 6, 802–813. 10.1038/ismej.2011.151 22094342 PMC3309359

[ref9] Bragina A. Berg C. Müller H. Moser D. Berg G. (2013). Insights into functional bacterial diversity and its effects on Alpine bog ecosystem functioning. Sci. Rep. 3, 1955–1962. 10.1038/srep01955 23739741 PMC6504810

[ref10] Bragina A. Oberauner-Wappis L. Zachow C. Halwachs B. Thallinger G. G. Müller H.. (2014). The *Sphagnum* microbiome supports bog ecosystem functioning under extreme conditions. Mol. Ecol. 23, 4498–4510. 10.1111/mec.12885, PMID: 25113243

[ref11] Bragina A. Stefanie M. Christian B. Henry M. Vladimir C. Franz H. (2012a). Similar diversity of alphaproteobacteria and nitrogenase gene amplicons on two related *Sphagnum* mosses. Front. Microbiol. 2, 275–284. 10.3389/fmicb.2011.00275 22294982 PMC3261640

[ref12] Brune A. Frenzel P. Cypionka H. (2000). Life at the oxic–anoxic interface: microbial activities and adaptations. FEMS Microbiol. Rev. 24, 691–710. 10.1111/j.1574-6976.2000.tb00567.x, PMID: 11077159

[ref13] Cai M. Hu C. Wang X. Zhao Y. Jia W. Sun X.. (2019). Selenium induces changes of rhizosphere bacterial characteristics and enzyme activities affecting chromium/selenium uptake by pak choi (*Brassica campestris* L. ssp. *Chinenis* Makino) in chromium contaminated soil. Environ. Pollut. 249, 716–727. 10.1016/j.envpol.2019.03.079, PMID: 30933769

[ref14] Caporaso J. G. Kuczynski J. Stombaugh J. Bittinger K. Bushman F. D. Costello E. K.. (2010). QIIME allows analysis of high-throughput community sequencing data. Nat. Methods 7, 335–336. 10.1038/nmeth.f.303, PMID: 20383131 PMC3156573

[ref15] Chambers L. G. Guevara R. Boyer J. N. Troxler T. G. Davis S. E. (2016). Effects of salinity and inundation on microbial community structure and function in a mangrove peat soil. Wetlands 36, 361–371. 10.1007/s13157-016-0745-8

[ref16] Cole J. R. Wang Q. Cardenas E. Fish J. Chai B. Farris R. J.. (2009). The ribosomal database project: improved alignments and new tools for rRNA analysis. Nucleic Acids Res. 37, D141–D145. 10.1093/nar/gkn879, PMID: 19004872 PMC2686447

[ref17] Daffonchio D. Borin S. Brusa T. Brusetti L. van der Wielen P. W. J. J. Bolhuis H.. (2006). Stratified prokaryote network in the oxic–anoxic transition of a deep-sea halocline. Nature 440, 203–207. 10.1038/nature04418, PMID: 16525471

[ref18] Danilova O. V. Belova S. E. Gagarinova I. V. Dedysh S. N. (2016). Microbial community composition and methanotroph diversity of a subarctic wetland in Russia. Microbiology 85, 545–554. 10.1134/S0026261716050039 29364602

[ref19] Dedysh S. N. (2011). Cultivating uncultured bacteria from northern wetlands: knowledge gained and remaining gaps. Front. Microbiol. 2, 184–198. 10.3389/fmicb.2011.00184, PMID: 21954394 PMC3174395

[ref20] Edgar R. C. (2010). Search and clustering orders of magnitude faster than BLAST. Bioinformatics 26, 2460–2461. 10.1093/bioinformatics/btq461, PMID: 20709691

[ref21] Edgar R. C. Haas B. J. Clemente J. C. Quince C. Knight R. (2011). UCHIME improves sensitivity and speed of chimera detection. Bioinformatics 27, 2194–2200. 10.1093/bioinformatics/btr381, PMID: 21700674 PMC3150044

[ref22] Elliott D. R. Caporn S. J. M. Nwaishi F. Nilsson R. H. Sen R. (2015). Bacterial and fungal communities in a degraded ombrotrophic peatland undergoing natural and managed re-vegetation. PLoS One 10, e0124726–e0124735. 10.1371/journal.pone.0124726, PMID: 25969988 PMC4430338

[ref23] Fierer N. Bradford M. A. Jackson R. B. (2007). Toward an ecological classification of soil bacteria. Ecology 88, 1354–1364. 10.1890/05-1839, PMID: 17601128

[ref24] Gilbert D. Mitchell E. A. D. (2006). “Microbial diversity in *Sphagnum* peatlands” in Peatlands: Evolution and records of environmental and climate changes. eds. Martini I. P. Cortizas A. M. Chesworth W. (Amsterdam: Elsevier), 287–318.

[ref25] Griffiths R. I. Thomson B. C. Plassart P. Gweon H. S. Stone D. Creamer R. E. (2016). Mapping and validating predictions of soil bacterial biodiversity using European and national scale datasets. Appl. Soil Ecol. 97, 61–68. 10.1016/j.apsoil.2015.06.018

[ref26] Hartman W. H. Richardson C. J. Vilgalys R. Bruland G. L. (2008). Environmental and anthropogenic controls over bacterial communities in wetland soils. Proc. Natl. Acad. Sci. USA 105, 17842–17847. 10.1073/pnas.0808254105 19004771 PMC2584698

[ref27] Hiraishi A. Shimada K. (2001). Aerobic anoxygenic photosynthetic bacteria with zinc-bacteriochlorophyll. J. Gen. Appl. Microbiol. 47, 161–180. 10.2323/jgam.47.161, PMID: 12483616

[ref28] Horwáth W. R. Kessel C. V. (2001). Acid fumigation of soils to remove carbonates prior to total carbon or carbon-13 isotopic analysis. Soil Sci. Soc. Am. J. 65, 1853–1856. 10.2136/sssaj2001.1853

[ref29] Huang X. Meyers P. A. Yu J. Wang X. Huang J. Jin F. (2012). Moisture conditions during the Younger Dryas and the early Holocene in the middle reaches of the Yangtze River, central China. The Holocene 22, 1473–1479. 10.1177/0959683612450202

[ref30] Huang X. Xue J. Wang X. Meyers P. A. Huang J. Xie S. (2013). Paleoclimate influence on early diagenesis of plant triterpenes in the Dajiuhu peatland, central China. Geochim. Cosmochim. Acta 123, 106–119. 10.1016/j.gca.2013.09.017

[ref31] Jaatinen K. Fritze H. Laine J. Laiho R. (2007). Effects of short- and long-term water-level drawdown on the populations and activity of aerobic decomposers in a boreal peatland. Glob. Chang. Biol. 13, 491–510. 10.1111/j.1365-2486.2006.01312.x

[ref32] Jackson C. R. Kong C. L. Yule C. M. (2009). Structural and functional changes with depth in microbial communities in a tropical Malaysian peat swamp forest. Microb. Ecol. 57, 402–412. 10.1007/s00248-008-9409-4, PMID: 18548182

[ref33] Jassey V. E. Gilbert D. Binet P. Toussaint M. L. Chiapusio G. (2011). Effect of a temperature gradient on *Sphagnum fallax* and its associated living microbial communities: a study under controlled conditions. Can. J. Microbiol. 57, 226–235. 10.1139/W10-116, PMID: 21358764

[ref34] Kanokratana P. Uengwetwanit T. Rattanachomsri U. Bunterngsook B. Nimchua T. Tangphatsornruang S.. (2011). Insights into the phylogeny and metabolic potential of a primary tropical peat swamp forest microbial community by metagenomic analysis. Microb. Ecol. 61, 518–528. 10.1007/s00248-010-9766-7, PMID: 21057783

[ref35] Karimi B. Terrat S. Dequiedt S. Saby N. Horrigue W. Lelièvre M.. (2018). Biogeography of soil bacteria and archaea across France. Sci. Adv. 4, eaat1808–eaat1821. 10.1126/sciadv.aat1808, PMID: 29978046 PMC6031370

[ref36] Killham K. Prosser J. I. (2015). “The bacteria and Archaea” in Soil microbiology, ecology and biochemistry. 4th Edn. ed. Paul E. A. (Boston: Academic Press), 41–76.

[ref37] Kip N. Fritz C. Langelaan E. S. Pan Y. (2012). Methanotrophic activity and diversity in different *Sphagnum magellanicum* dominated habitats in the southernmost peat bogs of Patagonia. Biogeosciences 9, 47–55. 10.5194/bg-9-47-2012

[ref38] Kip N. Winden J. F. V. Pan Y. Bodrossy L. Reichart G. J. Smolders A. J. P. (2010). Global prevalence of methane oxidation by symbiotic bacteria in peat-moss ecosystems. Nat. Geosci. 3, 617–621. 10.1038/ngeo939

[ref39] Koch I. H. Frederic G. Dunfield P. F. Overmann J. (2008). *Edaphobacter modestus* gen. nov., sp. nov., and *Edaphobacter aggregans* sp. nov., acidobacteria isolated from alpine and forest soils. Int. J. Syst. Evol. Microbiol. 58, 1114–1122. 10.1099/ijs.0.65303-0, PMID: 18450699

[ref40] Kostka J. E. Weston D. J. Glass J. B. Lilleskov E. A. Shaw A. J. Turetsky M. R. (2016). The *Sphagnum* microbiome: new insights from an ancient plant lineage. New Phytol. 211, 57–64. 10.1111/nph.13993, PMID: 27173909

[ref41] Kraigher B. Stres B. Hacin J. Ausec L. Mahne I. Jdvan E. (2006). Microbial activity and community structure in two drained fen soils in the Ljubljana Marsh. Soil Biol. Biochem. 38, 2762–2771. 10.1016/j.soilbio.2006.04.031

[ref42] Kulichevskaia I. S. Pankratov T. A. Dedysh S. N. (2006). Detection of representatives of the Planctomycetes in *Sphagnum* peat bogs by molecular and cultivation methods. Microbiology 75, 329–335. 10.1134/S0026261706030155 16871807

[ref43] Kwon M. J. Haraguchi A. Kang H. (2013). Long-term water regime differentiates changes in decomposition and microbial properties in tropical peat soils exposed to the short-term drought. Soil Biol. Biochem. 60, 33–44. 10.1016/j.soilbio.2013.01.023

[ref44] Lauber C. L. Hamady M. Knight R. Fierer N. (2009). Pyrosequencing-based assessment of soil pH as a predictor of soil bacterial community structure at the continental scale. Appl. Environ. Microbiol. 75, 5111–5120. 10.1128/AEM.00335-09, PMID: 19502440 PMC2725504

[ref45] Leff J. W. Jones S. E. Prober S. M. Barberán A. Borer E. T. Firn J. L. (2015). Consistent responses of soil microbial communities to elevated nutrient inputs in grasslands across the globe. Proc. Natl. Acad. Sci. USA 112, 10967–10972. 10.1073/pnas.1508382112 26283343 PMC4568213

[ref46] Li Y. Ma C. Zhu C. Huang R. Zheng C. (2016). Historical anthropogenic contributions to mercury accumulation recorded by a peat core from Dajiuhu montane mire, central China. Environ. Pollut. 216, 332–339. 10.1016/j.envpol.2016.05.083, PMID: 27289528

[ref47] Lin X. Green S. Tfaily M. M. Prakash O. Konstantinidis K. T. Corbett J. E.. (2012). Microbial community structure and activity linked to contrasting biogeochemical gradients in bog and fen environments of the Glacial Lake Agassiz Peatland. Appl. Environ. Microbiol. 78, 7023–7031. 10.1128/AEM.01750-12, PMID: 22843538 PMC3457479

[ref48] Lin X. Tfaily M. M. Steinweg J. M. Chanton P. Esson K. (2014). Microbial community stratification linked to utilization of carbohydrates and phosphorus limitation in a boreal peatland at Marcell Experimental Forest, Minnesota, USA. Appl. Environ. Microbiol. 80, 3518–3530. 10.1128/AEM.00205-14, PMID: 24682300 PMC4018854

[ref49] Mishra S. Lee W. A. Hooijer A. Reuben S. Sudiana I. M. Idris A. (2014). Microbial and metabolic profiling reveal strong influence of water table and land-use patterns on classification of degraded tropical peatlands. Biogeosciences 11, 14009–14042. 10.5194/bg-11-1727-2014

[ref50] Mitsch W. J. Nahlik A. M. Mander Ü. Zhang L. Anderson C. J. Jørgensen S. E. (2013). Wetlands, carbon, and climate change. Landsc. Ecol. 28, 583–597. 10.1007/s10980-012-9758-8

[ref51] Okamura K. Kawai A. Wakao N. Yamada T. Hiraishi A. (2015). *Acidiphilium iwatense* sp. nov., isolated from an acid mine drainage treatment plant, and emendation of the genus *Acidiphilium*. Int. J. Syst. Evol. Microbiol. 65, 42–48. 10.1099/ijs.0.065052-0, PMID: 25273513

[ref52] Opelt K. Berg G. (2004). Diversity and antagonistic potential of bacteria associated with bryophytes from nutrient-poor habitats of the Baltic Sea Coast. Appl. Environ. Microbiol. 70, 6569–6579. 10.1128/AEM.70.11.6569-6579.2004, PMID: 15528520 PMC525113

[ref53] Opelt K. Berg C. Berg G. (2007a). The bryophyte genus *Sphagnum* is a reservoir for powerful and extraordinary antagonists and potentially facultative human pathogens. FEMS Microbiol. Ecol. 61, 38–53. 10.1111/j.1574-6941.2007.00323.x 17484734

[ref54] Opelt K. Berg C. Schönmann S. Eberl L. Berg G. (2007b). High specificity but contrasting biodiversity of *Sphagnum*-associated bacterial and plant communities in bog ecosystems independent of the geographical region. ISME J. 1, 502–516. 10.1038/ismej.2007.58 18043652

[ref55] Opelt K. Chobot V. Hadacek F. Schönmann S. Eberl L. Berg G. (2007c). Investigations of the structure and function of bacterial communities associated with *Sphagnum* mosses. Environ. Microbiol. 9, 2795–2809. 10.1111/j.1462-2920.2007.01391.x 17922763

[ref56] Pankratov T. A. Ivanova A. O. Dedysh S. N. Werner L. (2011). Bacterial populations and environmental factors controlling cellulose degradation in an acidic *Sphagnum* peat. Environ. Microbiol. 13, 1800–1814. 10.1111/j.1462-2920.2011.02491.x, PMID: 21564458

[ref57] Pankratov T. A. Serkebaeva Y. M. Kulichevskaya I. S. Liesack W. Dedysh S. N. (2008). Substrate-induced growth and isolation of Acidobacteria from acidic *Sphagnum* peat. ISME J. 2, 551–560. 10.1038/ismej.2008.7, PMID: 18309356

[ref58] Philippot L. Andersson S. G. Battin T. J. Prosser J. I. Schimel J. P. Whitman W. B.. (2010). The ecological coherence of high bacterial taxonomic ranks. Nat. Rev. Microbiol. 8, 523–529. 10.1038/nrmicro2367, PMID: 20531276

[ref59] Raghoebarsing A. A. Smolders A. J. Schmid M. C. Rijpstra W. I. Woltersarts M. Derksen J.. (2005). Methanotrophic symbionts provide carbon for photosynthesis in peat bogs. Nature 436, 1153–1156. 10.1038/nature03802, PMID: 16121180

[ref60] Rütting T. Boeckx P. Müller C. Klemedtsson L. (2011). Assessment of the importance of dissimilatory nitrate reduction to ammonium for the terrestrial nitrogen cycle. Biogeosciences 8, 1779–1791. 10.5194/bg-8-1779-2011

[ref61] Schloss P. D. Westcott S. L. Ryabin T. Hall J. R. Hartmann M. Hollister E. B.. (2009). Introducing mothur: open-source, platform-independent, community-supported software for describing and comparing microbial communities. Appl. Environ. Microbiol. 75, 7537–7541. 10.1128/AEM.01541-09, PMID: 19801464 PMC2786419

[ref62] Segata N. Izard J. Waldron L. Gevers D. Miropolsky L. Garrett W. S.. (2011). Metagenomic biomarker discovery and explanation. Genome Biol. 12, R60–R77. 10.1186/gb-2011-12-6-r60, PMID: 21702898 PMC3218848

[ref63] Shaw A. J. Cox C. J. Boles S. B. (2010). Global patterns in peatmoss biodiversity. Mol. Ecol. 12, 2553–2570. 10.1046/j.1365-294X.2003.01929.x 12969461

[ref64] Shcherbakov A. V. Bragina A. V. Kuzmina E. Y. Berg C. Muntyan A. N. Makarova N. M. (2013). Endophytic bacteria of *Sphagnum* mosses as promising objects of agricultural microbiology. Microbiology 82, 306–315. 10.1134/S0026261713030107 24466733

[ref65] Shi Y. Li Y. Xiang X. Sun R. Yang T. He D. (2018). Spatial scale affects the relative role of stochasticity versus determinism in soil bacterial communities in wheat fields across the North China Plain. Microbiome 6, 27–39. 10.1186/s40168-018-0409-4 29402331 PMC5799910

[ref66] Smit E. Leeflang P. Gommans S. van den Broek J. van Mil S. Wernars K. (2001). Diversity and seasonal fluctuations of the dominant members of the bacterial soil community in a wheat field as determined by cultivation and molecular methods. Appl. Environ. Microbiol. 67, 2284–2291. 10.1128/AEM.67.5.2284-2291.2001, PMID: 11319113 PMC92868

[ref67] Soudzilovskaia N. A. Cornelissen J. H. During H. J. van Logtestijn R. S. Lang S. I. Aerts R. (2010). Similar cation exchange capacities among bryophyte species refute a presumed mechanism of peatland acidification. Ecology 91, 2716–2726. 10.1890/09-2095.1, PMID: 20957965

[ref68] Urbanová Z. Bárta J. (2014). Microbial community composition and in silico predicted metabolic potential reflect biogeochemical gradients between distinct peatland types. FEMS Microbiol. Ecol. 90, 633–646. 10.1111/1574-6941.12422, PMID: 25195805

[ref69] Urbanová Z. Barta J. (2016). Effects of long-term drainage on microbial community composition vary between peatland types. Soil Biol. Biochem. 92, 16–26. 10.1016/j.soilbio.2015.09.017

[ref70] van den Elzen E. Ljl V. D. B. Van d. W. B. Fritz C. Sheppard L. J. Lpm L. (2017). Effects of airborne ammonium and nitrate pollution strongly differ in peat bogs, but symbiotic nitrogen fixation remains unaffected. Sci. Total Environ. 610–611, 732–740. 10.1016/j.scitotenv.2017.08.102 28822940

[ref71] Vile M. A. Wieder R. K. Živković T. Scott K. D. Vitt D. H. Hartsock J. A. (2014). N_2_-fixation by methanotrophs sustains carbon and nitrogen accumulation in pristine peatlands. Biogeochemistry 121, 317–328. 10.1007/s10533-014-0019-6

[ref72] Ward N. L. Challacombe J. F. Janssen P. H. Bernard H. Coutinho P. M. Martin W.. (2009). Three genomes from the phylum Acidobacteria provide insight into the lifestyles of these microorganisms in soils. Appl. Environ. Microbiol. 75, 2046–2056. 10.1128/AEM.02294-08, PMID: 19201974 PMC2663196

[ref73] Weston D. J. Timm C. M. Walker A. P. Gu L. Muchero W. Schmutz J.. (2015). *Sphagnum* physiology in the context of changing climate: emergent influences of genomics, modeling and host-microbiome interactions on understanding ecosystem function. Plant Cell Environ. 38, 1737–1751. 10.1111/pce.12458, PMID: 25266403

[ref74] Wu Y. Blodau C. (2013). PEATBOG: a biogeochemical model for analyzing coupled carbon and nitrogen dynamics in northern peatlands. Geosci. Model Dev. 6, 1173–1207. 10.5194/gmd-6-1173-2013

[ref75] Xiang X. Wang H. M. Gong L. F. Liu Q. (2014). Vertical variations and associated ecological function of bacterial communities from *Sphagnum* to underlying sediments in Dajiuhu Peatland. Sci. China Earth Sci. 57, 1013–1020. 10.1007/s11430-013-4752-9

[ref76] Xiang X. Wang R. Wang H. Gong L. Man B. Xu Y. (2017). Distribution of Bathyarchaeota communities across different terrestrial settings and their potential ecological functions. Sci. Rep. 7, 45028–45038. 10.1038/srep45028 28322330 PMC5359579

[ref77] Yu Z. Loisel J. Brosseau D. P. Beilman D. W. Hunt S. J. (2010). Global peatland dynamics since the last glacial maximum. Geophys. Res. Lett. 37, L13402–L13406. 10.1029/2010GL043584

[ref78] Yun Y. Wang H. Man B. Xiang X. Zhou J. Qiu X.. (2016). The relationship between pH and bacterial communities in a single karst ecosystem and its implication for soil acidification. Front. Microbiol. 7, 1955–1968. 10.3389/fmicb.2016.01955, PMID: 28018299 PMC5159436

[ref79] Zhang J. Jiao S. Lu Y. (2018). Biogeographic distribution of bacterial, archaeal and methanogenic communities and their associations with methanogenic capacity in Chinese wetlands. Sci. Total Environ. 622–623, 664–675. 10.1016/j.scitotenv.2017.11.279 29223893

[ref80] Zhong Q. Chen H. Liu L. He Y. Zhu D. Jiang L.. (2017). Water table drawdown shapes the depth-dependent variations in prokaryotic diversity and structure in Zoige peatlands. FEMS Microbiol. Ecol. 93:fix049. 10.1093/femsec/fix049, PMID: 28431045

