# Supplementary material for: Structural Variations of Bacterial Community Driven by Sphagnum Microhabitat Differentiation in a Subalpine Peatland
Source: Front Microbiol. 2019 Jul 24;10:1661. doi: 10.3389/fmicb.2019.01661 (PMC6667737; doi:10.3389/fmicb.2019.01661)
Supplement: Supplementary file 1 [file Data_Sheet_1.docx]

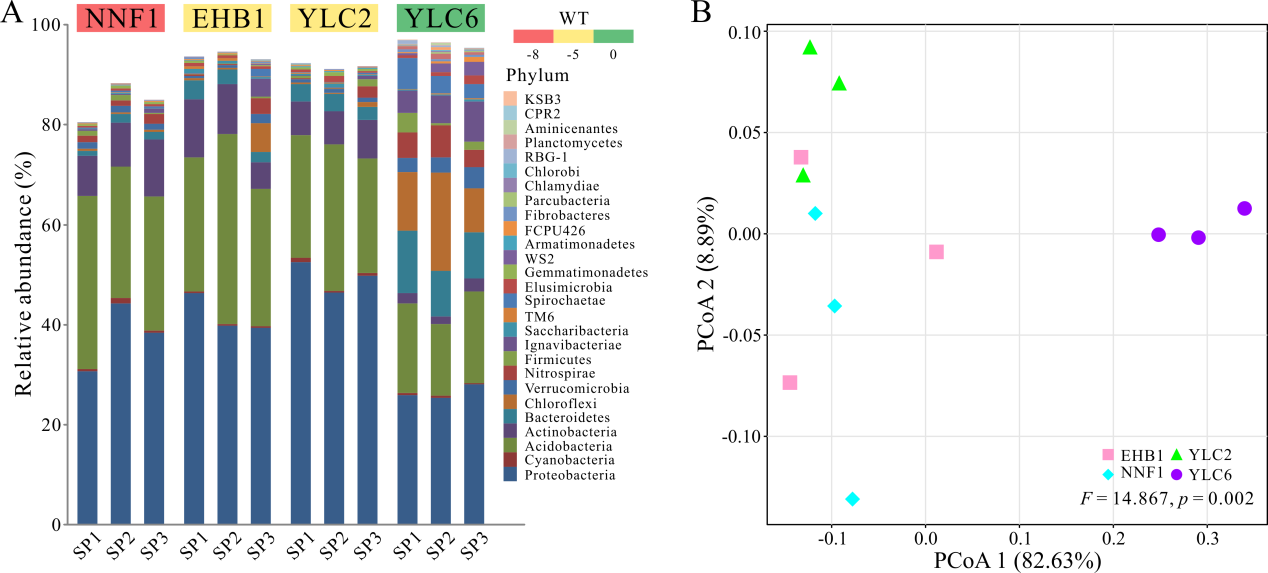


**Supplementary Figure S1.** Compositional **(A)** and structural differences **(B)** of bacterial community at phylum level in *S. palustre* peat samples. Site variations were calculated via PERMANOVA tests based on Bray-Curtis distances.

**Table S1.** Geochemical properties of *S. palustre* peat samples.

| **Parameter** | **EHB1-SP1** | **EHB1-SP2** | **EHB1-SP3** | **NNF1-SP1** | **NNF1-SP2** | **NNF1-SP3** | **YLC2-SP1** | **YLC2-SP2** | **YLC2-SP3** | **YLC6-SP1** | **YLC6-SP2** | **YLC6-SP3** |
| --- | --- | --- | --- | --- | --- | --- | --- | --- | --- | --- | --- | --- |
| F^-^[mg/kg] | 26.25 | 30.07 | 41.86 | 15.87 | 50.85 | 71.55 | 40.11 | 35.45 | 49.85 | 54.03 | 49.45 | 61.32 |
| Cl^-^[mg/kg] | 178.41 | 90.45 | 119.97 | 84.11 | 66.63 | 18.17 | 84.09 | 67.10 | 54.55 | 49.70 | 78.51 | 83.84 |
| NO_2_^-^[mg/kg] | 9.73 | 8.87 | 10.56 | 3.88 | 7.87 | 10.39 | 3.80 | 3.70 | 3.74 | 6.39 | 7.66 | 5.92 |
| SO_4_^2-^[mg/kg] | 175.49 | 156.35 | 160.39 | 52.59 | 99.43 | 57.68 | 163.11 | 138.41 | 154.76 | 251.67 | 249.06 | 165.82 |
| Br^-^[mg/kg] | 147.35 | 156.46 | 145.71 | 25.49 | 15.78 | 12.80 | 53.01 | 47.79 | 19.37 | 19.28 | 34.44 | 45.01 |
| NO_3_^-^[mg/kg] | 109.27 | 72.24 | 56.37 | 15.87 | 22.49 | 11.32 | 31.40 | 28.53 | 18.11 | 22.29 | 32.03 | 32.82 |
| PO_4_^3-^[mg/kg] | n.a. | n.a. | n.a. | n.a. | n.a. | n.a. | n.a. | n.a. | n.a. | n.a. | n.a. | n.a. |
| Li^+^[mg/kg] | 0.75 | n.a. | 0.78 | 0.28 | n.a. | n.a. | 0.37 | 0.37 | n.a. | 0.44 | n.a. | n.a. |
| Na^+^[mg/kg] | 41.42 | 29.58 | 46.79 | 18.10 | 11.64 | 11.03 | 8.18 | 38.73 | 48.91 | 36.78 | 40.94 | 31.48 |
| NH_4_^+^[mg/kg] | n.a. | n.a. | n.a. | n.a. | n.a. | n.a. | 50.32 | n.a. | n.a. | n.a. | n.a. | n.a. |
| K^+^[mg/kg] | 212.61 | 310.75 | 331.59 | 163.14 | 119.08 | 72.13 | 32.15 | 164.39 | 143.94 | 165.92 | 79.47 | 92.71 |
| Mg^2+^[mg/kg] | 26.51 | 32.72 | 46.36 | 53.25 | 51.01. | 99.79 | 5.39 | 13.97 | 12.56 | 18.45 | 14.63 | 15.51 |
| Ca^2+^[mg/kg] | 142.19 | 99.24 | 205.08 | 15.00 | 11.70. | 20.09 | 19.64 | 65.57 | 57.18 | 122.47 | 102.35 | 105.70 |
| TN[%] | 1.03 | 1.22 | 1.96 | 2.17 | 2.02 | 2.17 | 1.59 | 1.34 | 1.75 | 1.98 | 1.48 | 1.32 |
| TOC[%] | 39.42 | 40.03 | 39.12 | 27.99 | 31.50 | 28.78 | 37.16 | 36.96 | 34.19 | 42.05 | 39.90 | 45.02 |
| C/N ratio | 38.39 | 32.75 | 20.24 | 12.92 | 15.56 | 13.26 | 23.32 | 27.57 | 19.55 | 21.33 | 26.95 | 34.17 |
| Moisture[%] | 89.71 | 85.77 | 83.05 | 76.38 | 82.47 | 69.16 | 88.07 | 88.09 | 85.48 | 87.09 | 89.95 | 87.16 |
| OM[%] | 92.91 | 90.92 | 89.60 | 60.87 | 68.73 | 63.02 | 89.15 | 86.09 | 83.57 | 91.05 | 90.24 | 97.72 |
| pH | 5.60 | 5.71 | 5.64 | 6.70 | 6.54 | 6.60 | 5.94 | 5.81 | 5.9 | 5.47 | 5.35 | 5.51 |
| T/°C | 22.3 | 21.9 | 20.7 | 27.7 | 28.8 | 30.3 | 22.7 | 23.1 | 21.3 | 27.5 | 28.1 | 28.6 |
| WT/cm | -5 | -5 | -5 | -8 | -8 | -8 | -5 | -5 | -5 | 0 | 0 | 0 |

*EHB1: the first site of Erhaoba; NNF1: the first site of Niangniangfen; YLC2: the second site of Yangluchang; YLC6: the sixth site of Yangluchang. SP: S. palustre peat; TN: total nitrogen; TOC: total organic carbon; C/N: the ratio of total organic carbon to total nitrogen; OM: organic matter; WT: water Table. n.a.: not applied.*

**Table S2.** The number of reads in each sample before and after resampling.

| Reads | Befroe resample | After resample |  |
| --- | --- | --- | --- |
| EHB1-SP1 | 35063 | 29549 |  |
| EHB1-SP2 | 134134 | 29549 |  |
| EHB1-SP3 | 71305 | 29549 |  |
| EHB1-SB1 | 131733 | 29549 |  |
| EHB1-SB2 | 122481 | 29549 |  |
| EHB1-SB3 | 140064 | 29549 |  |
| EHB1-SG1 | 145433 | 29549 |  |
| EHB1-SG2 | 141022 | 29549 |  |
| EHB1-SG3 | 154775 | 29549 |  |
| NNF1-SP1 | 50206 | 29549 |  |
| NNF1-SP2 | 40952 | 29549 |  |
| NNF1-SP3 | 43886 | 29549 |  |
| NNF1-SB1 | 124832 | 29549 |  |
| NNF1-SB2 | 101274 | 29549 |  |
| NNF1-SB3 | 112369 | 29549 |  |
| NNF1-SG1 | 115578 | 29549 |  |
| NNF1-SG2 | 66435 | 29549 |  |
| EHB1-SG3 | 145390 | 29549 |  |
| YLC2-SP1 | 74096 | 29549 |  |
| YLC2-SP2 | 64089 | 29549 |  |
| YLC2-SP3 | 73308 | 29549 |  |
| YLC2-SB1 | 102055 | 29549 |  |
| YLC2-SB2 | 191602 | 29549 |  |
| YLC2-SB3 | 99872 | 29549 |  |
| YLC2-SG1 | 135181 | 29549 |  |
| YLC2-SG2 | 120963 | 29549 |  |
| YLC2-SG3 | 127933 | 29549 |  |
| YLC6-SP1 | 72626 | 29549 |  |
| YLC6-SP2 | 63802 | 29549 |  |
| YLC6-SP3 | 67139 | 29549 |  |
| YLC6-SB1 | 126579 | 29549 |  |
| YLC6-SB2 | 141265 | 29549 |  |
| YLC6-SB3 | 181810 | 29549 |  |
| YLC6-SG1 | 163144 | 29549 |  |
| YLC6-SG2 | 202350 | 29549 |  |
| YLC6-SG3 | 151946 | 29549 |  |

EHB1: the first site of Erhaoba; NNF1: the first site of Niangniangfen; YLC2: the second site of Yangluchang; YLC6: the sixth site of Yangluchang. SP: *S. palustre* peat; SB: *S. palustre* brown part; SG: *S. palustre* green part.

**Table S3.** ANOVA tests showing the differences of abundant taxa among different microhabitats.

| **Taxonomy** | **SP** | **SB** | **SG** | ***F*** | **Sig.** |
| --- | --- | --- | --- | --- | --- |
| Proteobacteria | 38.90 ± 9.46%^a^ | 48.92 ± 10.68%^a^ | 38.58 ± 15.93%^a^ | 2.719 | 0.081 |
| Alphaproteobacteria | 20.79 ± 6.00%^a^ | 23.14 ± 6.82%^a^ | 24.21 ± 10.28%^a^ | 0.583 | 0.564 |
| Gammaproteobacteria | 4.99 ± 3.18%^b^ | 17.66 ± 15.55%^a^ | 8.34 ± 9.87%^ab^ | 4.441 | 0.020 |
| Betaproteobacteria | 6.08 ± 3.74%^a^ | 4.32 ± 1.85%^ab^ | 2.68 ± 1.27%^b^ | 5.485 | 0.009 |
| Deltaproteobacteria | 6.17 ± 2.84%^a^ | 2.57 ± 1.89%^b^ | 0.62 ± 0.40^b^ | 24.216 | 0.000 |
| Cyanobacteria | 0.49 ± 0.22%^b^ | 9.61 ± 6.98%^b^ | 47.36 ± 21.05%^a^ | 45.188 | 0.000 |
| Cyanobacteria | 0.49 ± 0.22% ^b^ | 9.61 ± 6.98% ^b^ | 47.36 ± 21.05 ^a^ | 45.188 | 0.000 |
| Acidobacteria | 25.62 ± 6.76%^a^ | 16.20 ± 7.59%^b^ | 5.45 ± 2.35%^c^ | 33.686 | 0.000 |
| Acidobacteria | 25.62 ± 6.76%^a^ | 16.20 ± 7.59%^b^ | 5.45 ± 2.35%^c^ | 33.686 | 0.000 |
| Actinobacteria | 6.87 ± 3.47%^a^ | 7.55 ± 3.28%^a^ | 2.49 ± 1.59%^b^ | 10.707 | 0.000 |
| Actinobacteria | 6.87 ± 3.47%^a^ | 7.55 ± 3.28%^a^ | 2.49 ± 1.59%^b^ | 10.707 | 0.000 |

*SP: S. palustre peat; SB: S. palustre brown part; SG: S. palustre green par; F: statistical value, Sig., significant level (α = 0.05). Lowercase letters in the same row indicate differences among different microhabitats. Relative abundances decreased from SP to SG are highlighted in blue where those increase are in red.*

**Table S4**. Spearman's correlation of phyla (relative abundance > 1%) with environment factors.

| **Taxon** | **WT** | **TN** | **OM** | **Na^+^** | **Ca^2+^** | **Moisture** | **C/N** | **NO_3_^-^** | **pH** | **T** |
| --- | --- | --- | --- | --- | --- | --- | --- | --- | --- | --- |
| Proteobacteria | -0.376 | -0.221 | -0.245 | -0.007 | -0.315 | 0.056 | 0.070 | 0.077 | 0.448 | -0.483 |
| Acidobacteria | -0.751** | 0.186 | -0.524 | -0.301 | -0.357 | -0.517 | -0.280 | -0.112 | 0.678* | -0.105 |
| Actinobacteria | -0.751** | 0.018 | -0.329 | -0.287 | -0.350 | -0.392 | -0.126 | -0.035 | 0.608* | -0.021 |
| Bacteroidetes | 0.922** | -0.613* | 0.860** | 0.266 | 0.594* | 0.811** | 0.748** | 0.462 | -0.860** | -.0.056 |
| Chloroflexi | 0.683* | 0.011 | 0.434 | 0.517 | 0.608* | 0.287 | 0.098 | 0.028 | -0.678* | 0.042 |
| Nitrospirae | 0.512 | 0.249 | 0.259 | 0.357 | 0.413 | -0.042 | -0.154 | -0.126 | -0.531 | 0.217 |
| Ignavibacteriae | 0.478 | 0.287 | 0.231 | 0.168 | 0.322 | -0.084 | -0.168 | -0.161 | -0.434 | 0.336 |
| Verrucomicrobia | 0.410 | 0.357 | 0.112 | 0.091 | 0.189 | -0.105 | -0.224 | -0.210 | -0.364 | 0.441 |
| Spirochaetae | 0.478 | 0.326 | 0.182 | 0.287 | 0.364 | -0.105 | -0.196 | -0.238 | -0.490 | 0.329 |

*“*” and “**” represent significant correlation at the confidence interval 0.95 and 0.99, respectively. Abbreviations are indicated in* ***Table S1****.*

**Table S5.** One-way ANOVA of relative abundance of bacterial community across *S. palustre* peat samples.

| **Taxon** | **NNF1 (-8 cm)** | **EHB1 (-5 cm)** | **YLC2 (-5 cm)** | **YLC6 (0 cm)** | |
| --- | --- | --- | --- | --- | --- |
| Proteobacteria | 37.77 ± 6.86%^b^ | 41.83 ± 3.91%^ab^ | 49.57 ± 3.08%^a^ | 26.44 ± 1.43%^c^ | |
| **Alphaproteobacteria** | **23.36 ± 2.56%^a^** | **25.18 ± 2.49%^a^** | **23.17 ± 1.59%^a^** | **11.47 ± 2.25%^b^** | |
| Methylocystaceae | 0.15 ± 0.06%^a^ | 0.03 ± 0.02%^b^ | 0.04 ± 0.03%^b^ | 0.04 ± 0.03%^b^ | |
| **Gammaproteobacteria** | **4.29 ± 1.69%^ab^** | **6.16 ± 3.08%^ab^** | **8.29 ± 1.65%^a^** | **1.22 ± 0.17%^b^** |  |
| Methylococcaceae | 0.03 ± 0.02%^a^ | 0.5 ± 0.60%^a^ | 0.39 ± 0.46%^a^ | 0.03 ± 0.01%^a^ |  |
| Betaproteobacteria | 5.47 ± 1.80%^b^ | 4.52 ± 1.53%^b^ | 11.71 ± 0.86%^a^ | 2.63 ± 0.93%^b^ | |
| **Deltaproteobacteria** | **4.26 ± 0.86%^b^** | **4.91 ±2.15%^b^** | **5.16 ± 1.52%^b^** | **10.35 ± 1.02%^a^** | |
| Epsilonproteobacteria | 0.00 ± 0.00%^a^ | 0.01 ± 0.01%^a^ | 0.00 ± 0.00%^a^ | 0.02 ± 0.02%^a^ | |
| Cyanobacteria | 0.65 ± 0.28%^a^ | 0.33 ± 0.05%^a^ | 0.58 ± 0.28%^a^ | 0.39 ± 0.09%^a^ | |
| Acidobacteria | 29.25 ± 4.62%^a^ | 30.77 ± 6.28%^a^ | 25.61 ± 3.38%^ab^ | 16.86 ± 2.17%^b^ | |
| **Actinobacteria** | **9.43 ± 1.80%^a^** | **8.99 ± 3.23%^a^** | **7.04 ± 0.67%^a^** | **2.03 ± 0.56%^b^** | |
| Bacteroidetes | 1.41 ± 0.39%^b^ | 2.93 ± 0.92%^b^ | 3.18 ± 0.51%^b^ | 10.31 ± 1.92%^a^ | |
| **Chloroflexi** | **0.38 ± 0.09%^b^** | **2.16 ± 3.16%^b^** | **0.50 ± 0.35%^b^** | **13.40 ± 5.58%^a^** | |
| Verrucomicrobia | 1.27 ± 0.05%^b^ | 0.94 ± 0.76%^b^ | 0.77 ± 0.13%^b^ | 3.35 ± 0.72%^a^ | |
| Nitrospirae | 1.46 ± 0.39%^b^ | 1.27 ± 1.69%^b^ | 0.86 ± 1.24%^b^ | 5.04 ± 1.44%^a^ | |
| Firmicutes | 0.74 ± 0.45%^a^ | 0.15 ± 0.13%^a^ | 0.68 ± 0.68%^a^ | 1.98 ± 1.76%^a^ | |
| Ignavibacteriae | 0.50 ± 0.36%^b^ | 1.25 ± 2.05%^b^ | 0.25 ± 0.26%^b^ | 5.97 ± 1.84%^a^ | |
| Saccharibacteria | 0.12 ± 0.06%^a^ | 0.58 ± 0.32%^a^ | 0.44 ± 0.27%^a^ | 0.29 ± 0.10%^a^ | |
| TM6 | 0.07 ± 0.08%^a^ | 0.36 ± 0.21%^a^ | 0.19 ± 0.11%^a^ | 0.17 ± 0.07%^a^ | |
| Spirochaetae | 0.25 ± 0.08%^b^ | 0.55 ± 0.83%^b^ | 0.15 ± 0.12%^b^ | 4.22 ± 1.78%^a^ | |
| Elusimicrobia | 0.41 ± 0.03%^a^ | 0.54 ± 0.08%^a^ | 0.71 ± 0.42%^a^ | 0.98 ± 0.61%^a^ | |
| Gemmatimonadetes | 0.41 ± 0.13%^a^ | 0.31 ± 0.09%^ab^ | 0.56 ± 0.21%^a^ | 0.02 ± 0.01%^b^ | |
| WS2 | 0.00 ± 0.00%^b^ | 0.06 ± 0.07%^b^ | 0.00 ± 0.00%^b^ | 1.58 ± 1.16%^a^ | |
| Armatimonadetes | 0.07 ± 0.03%^b^ | 0.07 ± 0.01%^b^ | 0.16 ± 0.03%^a^ | 0.03 ± 0.02%^b^ | |
| FCPU426 | 0.19 ± 0.01%^a^ | 0.17 ± 0.06%^a^ | 0.22 ± 0.08%^a^ | 0.53 ± 0.39%^a^ | |
| Fibrobacteres | 0.04 ± 0.03%^b^ | 0.19 ± 0.10%^b^ | 0.06 ± 0.00%^b^ | 0.48 ± 0.08%^a^ | |
| Parcubacteria | 0.01 ± 0.01%^b^ | 0.07 ± 0.02%^ab^ | 0.06 ± 0.06%^ab^ | 0.23 ±0.14%^a^ | |
| Chlamydiae | 0.01 ± 0.01%^b^ | 0.02 ± 0.02%^ab^ | 0.01 ± 0.01%^b^ | 0.11 ± 0.06%^a^ | |
| Chlorobi | 0.03 ± 0.01%^b^ | 0.05 ± 0.08%^ab^ | 0.01 ± 0.01%^b^ | 0.21 ± 0.11%^a^ | |
| RBG-1 | 0.00 ± 0.00%^a^ | 0.00 ± 0.00%^a^ | 0.00 ± 0.00%^a^ | 0.33 ± 0.35%^a^ | |
| Planctomycetes | 0.01 ± 0.01%^a^ | 0.01 ± 0.02%^a^ | 0.01 ± 0.01%^a^ | 0.07 ± 0.05%^a^ | |
| Aminicenantes | 0.00 ± 0.00%^a^ | 0.00 ± 0.00%^a^ | 0.00 ± 0.00%^a^ | 0.14 ± 0.24%^a^ | |
| CPR2 | 0.00 ± 0.00%^a^ | 0.03 ± 0.06%^a^ | 0.00 ± 0.00%^a^ | 0.01 ± 0.00%^a^ | |
| KSB3 | 0.00 ± 0.00%^a^ | 0.00 ± 0.00%^a^ | 0.00 ± 0.00%^a^ | 0.01 ± 0.01%^a^ | |

*Values are means ± standard error. Lowercase letters in the same row indicate significant differences at P < 0.05. Values in the parenthesis are the water table.*
